# Ultrasonographic Applications of Novel Technologies and Artificial Intelligence in Critically Ill Patients

**DOI:** 10.3390/jpm14030286

**Published:** 2024-03-07

**Authors:** Sławomir Mika, Wojciech Gola, Monika Gil-Mika, Mateusz Wilk, Hanna Misiolłek

**Affiliations:** 1 Medica Co., Ltd. (Upper Silesian School of Ultrasonography), 41-500 Chorzów, Poland; 2 Collegium Medicum, Jan Kochanowski University (JKU), 25-317 Kielce, Poland; wojciech.gola@ujk.edu.pl; 3 Municipal Hospital Co., Ltd., 41-703 Ruda Śląska, Poland; mmika@szpitalruda.pl; 4 Collegium Medicum, WSB University, 41-300 Dąbrowa Górnicza, Poland; mateusz.wilk@wsb.edu.pl; 5 Department of Anaesthesiology and Critical Care, School of Medicine with the Division of Dentistry, Medical University of Silesia, 41-808 Zabrze, Poland; hanna.misiolek@sum.edu.pl

**Keywords:** artificial intelligence, ultrasonography, intensive care

## Abstract

The diagnostic process in Intensive Care Units has been revolutionized by ultrasonography and accelerated by artificial intelligence. Patients in critical condition are often sonoanatomically challenging, with time constraints being an additional stress factor. In this paper, we describe the technology behind the development of AI systems to support diagnostic ultrasound in intensive care units. Among the AI-based solutions, the focus was placed on systems supporting cardiac ultrasound, such as Smart-VTI, Auto-VTI, SmartEcho Vue, AutoEF, Us2.ai, and Real Time EF. Solutions to assist hemodynamic assessment based on the evaluation of the inferior vena cava, such as Smart-IVC or Auto-IVC, as well as to facilitate ultrasound assessment of the lungs, such as Smart B-line or Auto B-line, and to help in the estimation of gastric contents, such as Auto Gastric Antrum, were also discussed. All these solutions provide doctors with support by making it easier to obtain appropriate diagnostically correct ultrasound images by automatically performing time-consuming measurements and enabling real-time analysis of the obtained data. Artificial intelligence will most likely be used in the future to create advanced systems facilitating the diagnostic and therapeutic process in intensive care units.

## 1. Introduction

Ultrasound has a particular application in intensive care. Since the sonoanatomical conditions in patients treated in intensive care units (ICU) are far from ideal, and ultrasound (US) imaging is often performed under time pressure, physicians are increasingly more likely to use artificial intelligence (AI). In particular, the use of AI in intensive care has been discussed very often in both the literature and the media in the last 2–3 years. Modern technologies have led to progress in the development of medical equipment dedicated to anesthesiology and intensive care [1]. AI has been widely used in both these fields, making it significantly easier to interpret the obtained images and providing doctors with rapid feedback on the patient’s clinical situation [2,3].

Point-of-care ultrasound (POCUS) has a wide diagnostic application in intensive care, making it easier to use and interpret such imaging modalities as transthoracic and transesophageal echocardiography, lung ultrasound, ultrasound of a patient in shock, and patients with other life-threatening conditions. Due to the complexity of ultrasound assessment in intensive care, the physician’s purpose of using AI in this setting is to help assess the patient’s condition based on US imaging of various systems and organs and assess the patient’s hemodynamic profile [4,5,6]. The literature has shown that AI-guided POCUS will most likely increase the efficiency and effectiveness of ultrasound imaging [4,7]. AI-supported point-of-care protocols also have particular educational applications, enabling young medical students to improve ultrasound techniques and increasing the identifiability of anatomical structures, which is necessary to perform procedures [8].

Excellent knowledge of normal, topographic, and pathological anatomy is essential for a correct assessment of ultrasound images. High inter-patient anatomical variability and often difficult sonoanatomical conditions may discourage less experienced anesthesiologists from performing US [3]. Point-of-care protocols require excellent technique in operating an ultrasonic transducer from the operator due to time constraints and the schematic nature of this type of examination.

In this complex setting, AI comes to the aid of clinicians [9]. Depending on the type of software, it helps detect key structures for performing a specific medical procedure, such as cannulation, finding the optimal ultrasound plane for imaging of the heart, lungs, or abdominal organs [4,10] as well as setting the appropriate image quality, and performing measurements, including complex Doppler measurements [11]. At the same time, it significantly reduces the time needed to learn how to perform imaging and the time to acquire clinically significant images, and it improves the accuracy of the obtained results [2,10,12,13].

## 2. Selected Innovative AI-Based US Technologies and Solutions Intended for Critically Ill Patients

Intensive care ultrasonography (POCUS) is a tool that can provide abundant clinically relevant information about a patient’s condition. The doctors attending the patient change, which, combined with the diversity of medical staff in terms of ultrasound imaging skills, may lead to variable and often incomplete observations and conclusions. A typical POC examination is often performed under time pressure. AI-based solutions prove extremely helpful in this regard and are very dynamically entering everyday clinical practice. They allow for obtaining more objective real-time results than those obtained by different operators [14]. Similar to regional anesthesiology, these solutions are based on machine learning (ML), deep learning (DL) and deep convolutional neutral network (DCNN), U-Net, and big data. These are information technologies used in the processing and very detailed interpretation of images, including medical images. In the case of DL, the created software is intended to imitate and simulate human thinking in terms of the processed information and is an important component of ML, which together constitute artificial intelligence. ML, in turn, is the machine’s ability to automatically learn and improve the interpretation of images [15]. The key technologies discussed in this paper include the following.

**Figure 1:**
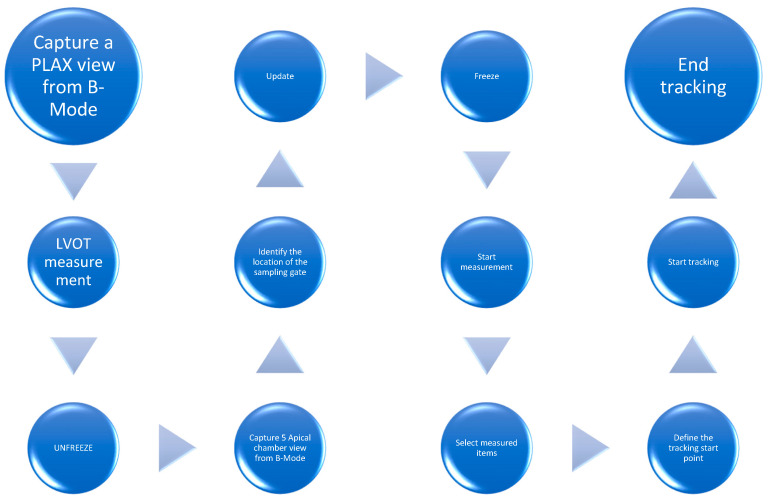
Manual VTI measurement.

SmartVTI (Mindray Medical International Limited, Shenzhen, China) hereinafter Mindray), AutoVTI (General Electric, Boston, MA, USA), US2.AI. Cardiac Output Workflow (EchoNous Inc., Redmond Washington, DC, USA) or LVivo Seamless (Philips Medical Systems International, Best, Holandia) are examples of AI-based tools offered by two different manufacturers, which are used in ultrasound of patients in a life-threatening condition. These semi-automatic solutions make it possible to calculate two key parameters for hemodynamic monitoring and provide important prognostic data for a critically ill patient. These two parameters are the automatic measurement of stroke volume (SV) in mL and the cardiac output (CO), which is the product of the heart rate (HR) and the SV [15,16,17]. Typical and manual examination of these two measurements requires at least two ultrasound views (PLAX—parasternal long axis view), as well as LVOT (left ventricular outflow tract) measurement, and Doppler spectrum contouring by setting the sampling gate parallel to the LVOT blood flow from the five-chamber view (5CH). The inter-operator results may differ due to incorrect placement of the Doppler gate and incorrect selection of the insonation angle, which may consequently lead to overestimation of the velocity time integral (VTI) [18]. Considering the fact that the SV is the product of LVOT and VTI, the final value of the stroke volume may vary significantly depending on the operator’s skill and precision. The sequence of steps in the manual approach is shown in Figure 1.

**Figure 2:**
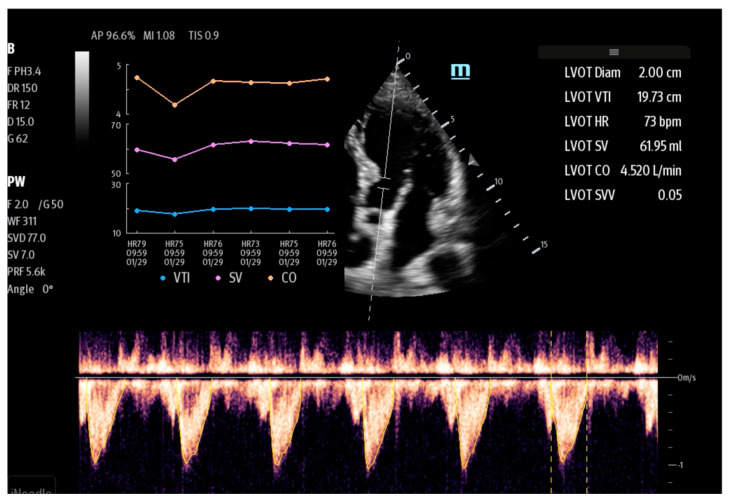
SmartVTI and a trending graph (Mindray TEX20; software version 01(01.07.00)).

When using AI-based tools, the operator should perform only one manual measurement, i.e., LVOT in the PLAX view. The second and last step is to obtain a 5CH view and, through “one-click” (SmartVTI for Mindray or AutoVTI for GE devices), the device will automatically set the Doppler gate in the left ventricular outflow tract (LVOT) and outline the Doppler spectrum by tracing the velocity in order to calculate the VTI in real time. With data in the form of the LVOT area and VTI as well as the heart rate (HR), the ultrasound device is able to automatically calculate the SV and CO parameters in real time, presenting the data in the form of trending graphs (Figure 2).

The calculations consist of simple mathematical operations using the following formulas [19]:

SV (mL/cycle) = LVOT Area (cm^2^) × VTI/cycle (cm/cycle)

CO (L/min) = SV (L/cycle) × HR (cycle/min)

**Figure 3:**
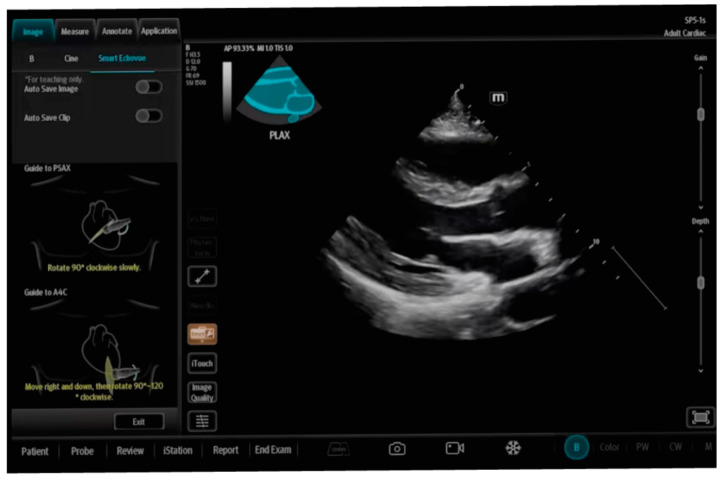
SmartEchoVue (Mindray TEX20; software version 01(01.07.00)).

Additionally, some manufacturers of ultrasound equipment have access to technology that allows the assessment of views obtained by the operator during an examination. They bear in mind the fact that the above measurements are reliable only when appropriate PLAX and 5CH images are obtained, and the device provides the operator with a graphic “prompt” on whether the obtained image is acceptable or whether it should be corrected. By properly selecting colors, the operator knows whether a given view is appropriate or whether it requires more involvement. Only when the obtained images are accepted by the AI algorithm are the automatic measurements taken [20]. An example of SmartEchoVue (Mindray), Us2.ai and AI TRO (EchoNous Inc.), Butterfly ScanLab (Butterfly Network, Inc.) or LVivo Seamless (Philips) technology enabling the correction of appropriate views is shown in Figure 3.

**Figure 4:**
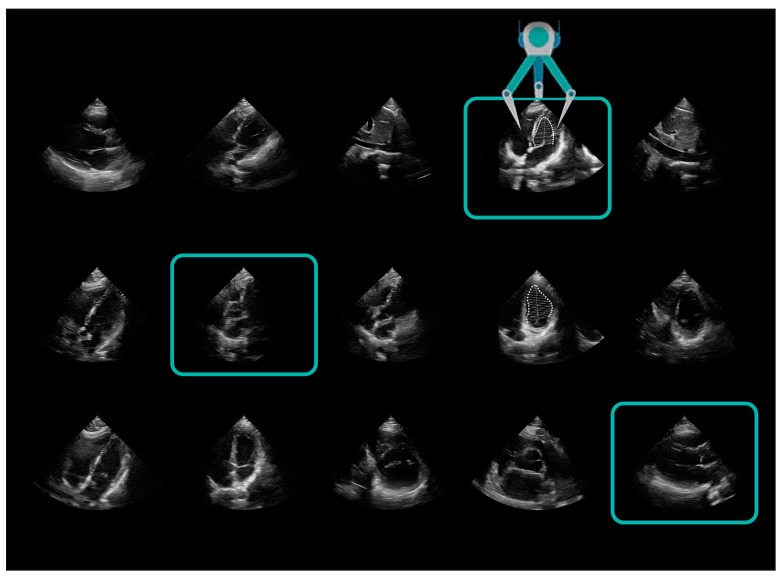
LVivo Seamless (Philips Epiq Elite, software version 9.05).

In the case of solutions proposed by Philips in the LVivo Seamless technology, the device itself selects the most optimal projections obtained during an ultrasound scan and uses them to present cardiac measurements, including SV [21] (Figure 4).

**Figure 5:**
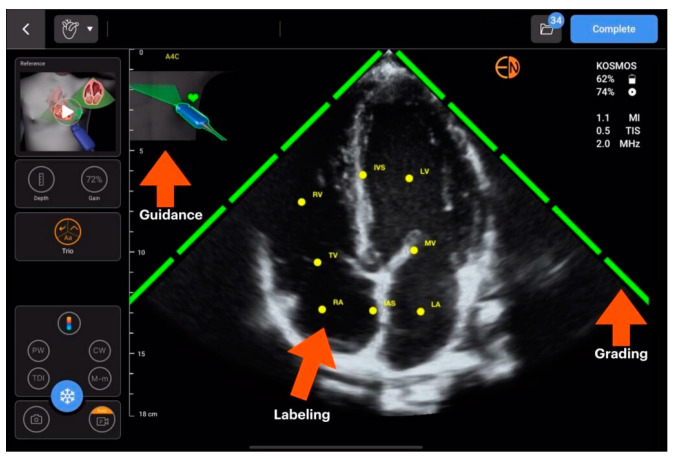
AI Trio (EchoNous, Inc.), adapted from (source: https://echonous.com/product/kosmos-ai/) with permission publisher 2024 (accessed on 1 January 2024) [22].

**Figure 6:**
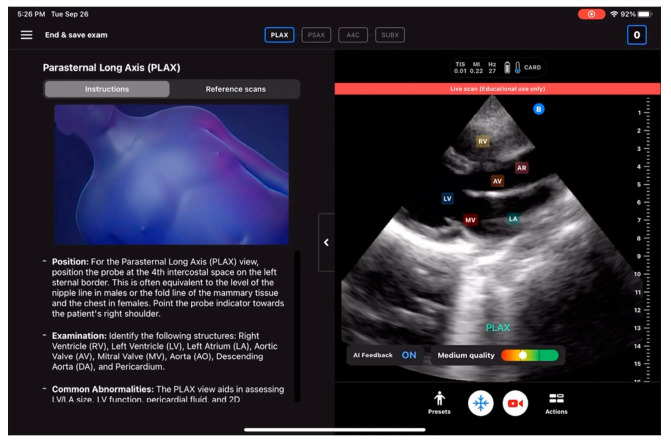
Butterfly ScanLab (Butterfly Network, Inc.), adapted from (https://www.butterflynetwork.com/education) with permission publisher 2024 (accessed on 1 January 2024) [23].

AI TRIO (EchoNous, Inc.) and Butterfly ScanLab, which offer real-time support during an ultrasound scan, are yet another solution implemented by EchoNous, Inc., and Butterfly Network, Inc. (Boston, MA, USA), which allows even beginners to quickly make progress in performing echocardiography. They help the operator to identify quickly and very accurately individual cardiac structures and assess the quality of and initially qualify the obtained images. This solution is presented in Figure 5 and Figure 6.

**Figure 7:**
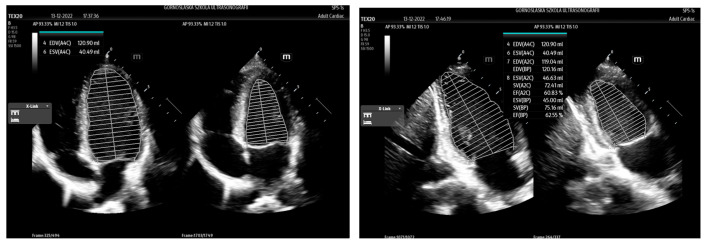
EF measurement on 4CH and 2 CH (Mindray TEX20; software version 01(01.07.00)).

AutoEF (Mindray)/Real Time EF (GE)/ Us2.ai (EchoNous Inc.)/LVivo EF (Philips) is another tool to assist clinicians in their work, allowing the calculation of the ejection fraction (EF) in real time based on AI. The assessment of the left ventricular (LV) EF is an integral part of echocardiographic evaluation. Despite not being error-free, it is one of the basic criteria for therapeutic decisions and a prognostic marker. EF assessment allows for a quantitative assessment of the blood volume ejected from the left ventricle during each heartbeat. In the traditional and manual approach, it is a multi-stage process that includes both systolic and diastolic measurements of the left ventricle. Measurements can be made in two views: 4CH (four chamber view) and 2CH (two chamber view) as shown in Figure 7.

Based on the obtained measurements, the ultrasound machine uses the built-in calculation package to estimate the EF according to the following formula:

EF = (End Diastolic Volume − End Systolic Volume)/End Diastolic Volume × 100

**Figure 8:**
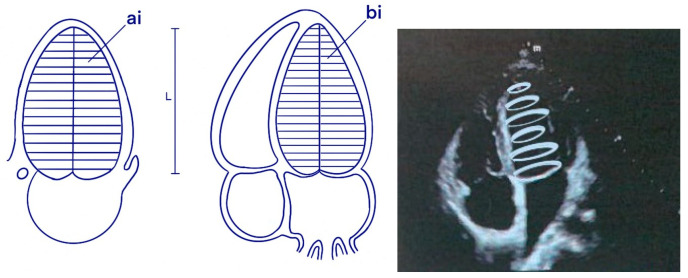
Method for calculating EF using the Simpson method.

This measurement is based on the Simpson method, i.e., the biplane method of disk summation as shown in Figure 8.

**Figure 9:**
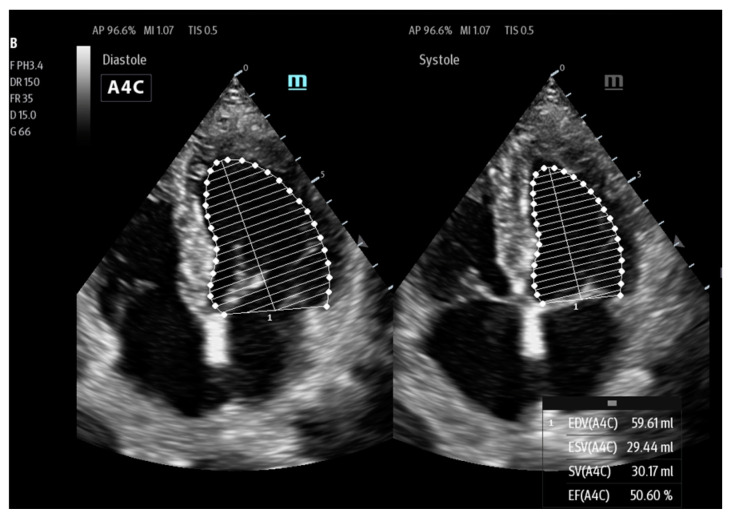
Automatic EF measurement (Mindray TEX20; software version 01(01.07.00)).

Manual outlining of the LV endocardium may be very time consuming and burdened by inter-operator measurement errors, not to mention the significant time needed to perform the scan itself. In the intensive care and according to the POCUS philosophy, often, only a quantitative method is used to assess global LV dysfunction and thereby the patient’s general status. AutoEF or RealTime EF may be a tool to solve many of the abovementioned problems related to EF assessment (Figure 9).

The operator’s main task is to obtain an apical four chamber view (A4CH). Using AI algorithms, ML and DL in particular, the ultrasound device first classifies the image obtained by the operator by tracing individual ventricular wall segments both in the end-diastolic and end-systolic phases, while assessing the maximum and minimum LV volume. If the view and the correct image have been accepted by the ultrasound machine, the most optimal views are selected in one cycle, and the LVEF is automatically calculated. The calculated parameters are displayed on the ultrasound screen. Each time, the operator has the opportunity to correct the outlined LV endocardium if necessary.

**Figure 10:**
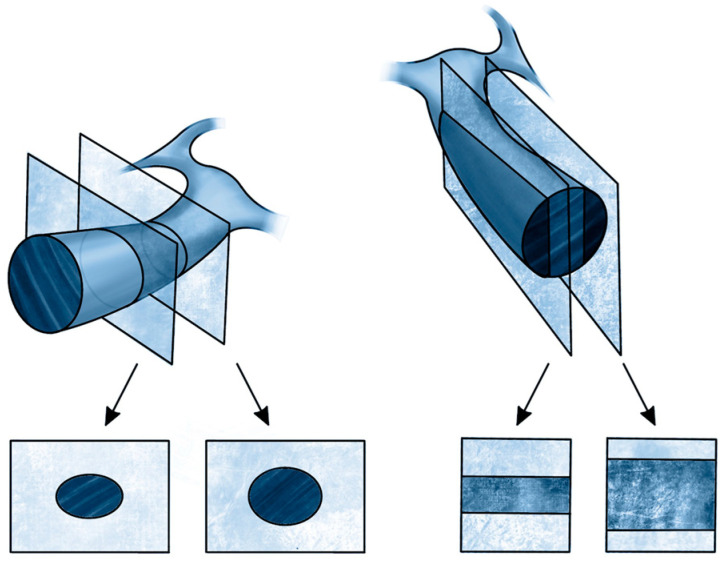
Imaging variability of the inferior vena cava depending on the respiratory phase.

The technology of the automatic measurement of the inferior vena cava (IVC), referred to by various manufacturers as SmartIVC (Mindray) and AutoIVC (GE), is an additional tool to support the patient’s assessment and deliver clinically relevant information (Figure 10). The IVC width and collapsibility are indirect indicators of myocardial preload and CVP estimation. Similar to LVEF measurement, ultrasonographic IVC assessment can also be highly subjective. The final dimension of the inferior vena cava is influenced by, among other things, the operator’s skill and, above all, the unstable position of this vessel during respiratory movements. During spontaneous breathing, the IVC moves on average 21.7 mm vertically and 3.9 mm horizontally. Furthermore, the actual vertical axis of the IVC in the human body is not 90° but an average of 115°. All this means that the test is not easy to interpret, and it should take into account these limitations [24,25].

**Figure 11:**
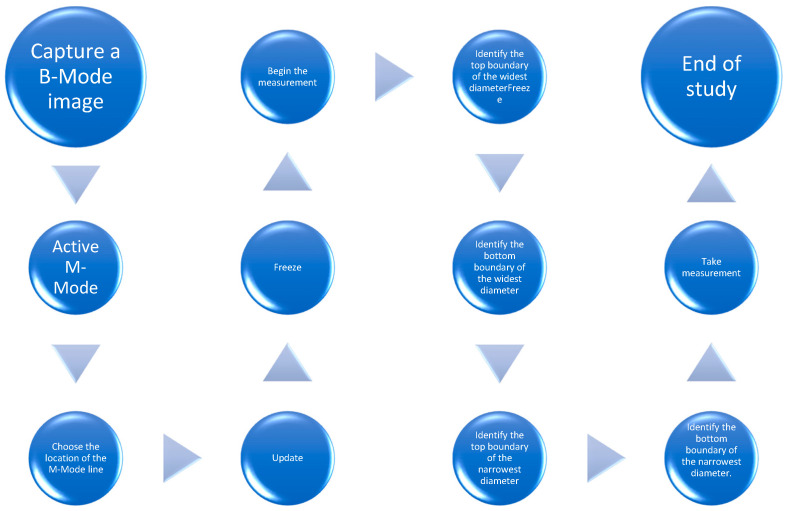
Conventional IVC measurement.

According to the 2015 guidelines of the American Society of Echocardiography (ASE) [26], the measurement should be performed with the patient in a supine position, as the IVC diameter and shape may change depending on the patient’s position, at a distance of 1–2 cm from the opening of the hepatic vein, perpendicular to the long axis of the vessel in B-Mode. In clinical practice, M-Mode is also used to observe IVC respiratory variability [24]. The traditional and manual diagnostic process is presented in Figure 11.

The semi-auto SmartIVC and AutoIVC provide quick and simple real-time IVC measurements without the need to manually perform the individual stages of the examination described in Figure 11.

After obtaining a correct view in the long axis of the IVC and activating the SmartIVC or AutoIVC button on the US control panel, the algorithm automatically determines the anatomical M-line, which is placed exactly at the right site, where the measurements are made manually. Both the angle and the position of the M-line are adjusted in real time using a special tracing algorithm. If the image changes due to the mobility of the IVC described earlier, the M-line automatically adjusts so that it is always perpendicular to IVC axis. The user can always adjust the position and angle of the anatomical M-line. Owing to the automatic detection of the IVC walls, the presented results show its maximum and minimum dimensions and the Caval Index (CI). It is calculated according to the following formula:

Caval Index (CI) = (Maximum diameter (expiratory) − Minimum diameter (inspiratory))/Maximum diameter (expiratory).

In the case of a mechanically ventilated patient, the DI (Distensibility Index) is presented instead of the CI, calculated according to the following formula:

Distensibility Index (DI) = (Maximum diameter (expiratory) − Minimum diameter (inspiratory))/Minimum diameter (inspiratory).

**Figure 12:**
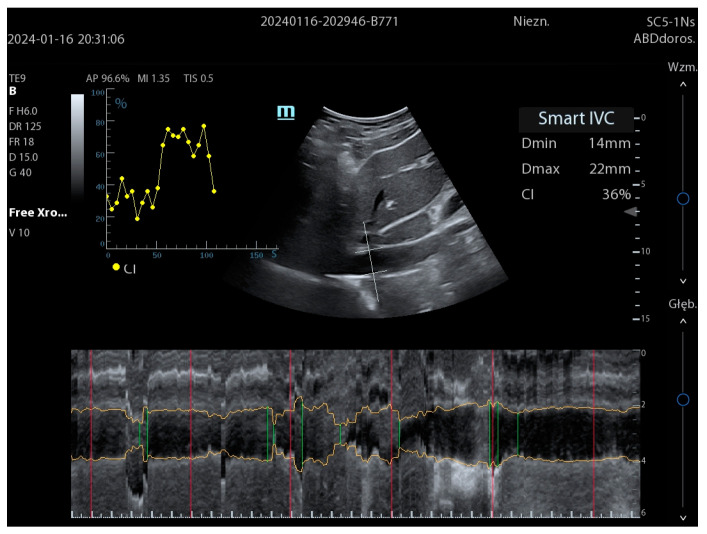
Auto-measurement of IVC using SmartIVC (Mindray TEX20; software version 01(01.07.00)).

Furthermore, in addition to the CI and DI, the technology allows for presenting the trend lines in accordance with the change in collapsibility, as shown in Figure 12.

The Inferior Vena Cava Distensibility Index (IVCDI) is a parameter commonly used in critical care units to assess the fluid responsiveness and intravascular volume status in ventilated patients. It involves measuring changes in the diameter of the inferior vena cava (IVC) during the respiratory cycle. The IVC in deeply sedated or paralyzed patients being passively ventilated with positive pressure has a very different pattern in respiratory variation compared to that of spontaneously breathing patients. In the absence of central hypovolemia, the increase in the internal IVC pressure that has been transmitted from the right atrium exceeds the increase in the external intra-abdominal pressure. This pressure gradient will distend the IVC; so, its size will increase on inspiration. This does not occur in a patient who is fluid unresponsive. When the caval venous system is congested and closer to its maximal size, there will be minimal, if any, distention of the IVC with inspiration. These phenomena can be quantified by the “IVC Distensibility Index”.

The Vena Cava Distensibility Index and distensibility variability can be calculated with the following formulas: IVC-DI = (max diameter–min diameter)/(min diameter) × 100, IVC-DV = [(maximum diameter − minimum diameter)/(mean diameter)] × 100). The cut off of the distensibility index for fluid responsiveness is between 15 and 20%, with maximum accuracy when Vt ≥ 8 mL/Kg or PEEP ≤ 5 cm H_2_O.

However, to reiterate, the change in IVC size with positive pressure mechanical ventilation is only observed consistently in patients who have no inspiratory effort and are being ventilated passively. These are patients who are either deeply sedated or paralyzed [27,28,29,30,31].

**Figure 13:**
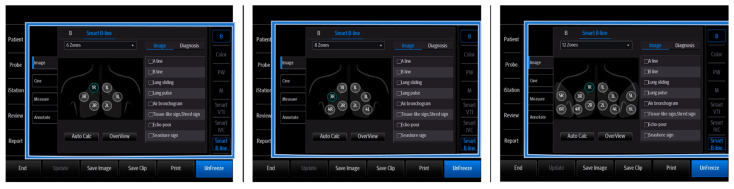
Zone annotation screen (Mindray TEX20; software version 01(01.07.00)).

Airway ultrasound is becoming a common, accessible, quick, and easy method for assessing the upper airways. Its main goal, especially in the case of intensive care patients, is to identify anatomical structures such as thyroid cartilage, epiglottis, cricoid cartilage, cricothyroid membrane, tracheal cartilages, and esophagus, determine the required size of endotracheal tubes (ETT), aid potentially difficult intubation, and help perform a percutaneous tracheostomy [32]. Research on the use of AI in airway ultrasound is currently underway, pointing to the enormous potential of this technology [32]. Due to its specificity, airway US is also related to lung ultrasound, in which AI is already used. In addition to the previously mentioned assessment of the myocardium and the inferior vena cava, AI technologies are also utilized to assess the respiratory system. Since 1992, when Professor Daniel Lichtenstein published an article on lung ultrasound, this discipline has become increasingly appreciated by clinicians and is increasingly used in critically ill patients. Lung ultrasound is used to interpret artifacts, the presence or absence of which may be of key importance in the clinical assessment. B-lines are one of the key findings assessed in lung ultrasound. The presence of these vertical artifacts excludes pneumothorax on the one hand but is a marker of interstitial fluid accumulation on the other hand. The new guidelines (February 2023) [33] for the use of lung ultrasound not only once again clearly confirm that AI has a wide application in many fields of medicine but also point to its powerful potential in lung diagnosis. Combining technology and diagnostic imaging may prove extremely useful as the first screening in an intensive care unit [33]. Conventional lung ultrasound focuses on the assessment of horizontal and vertical artifacts and involves their visual assessment. A convex transducer is used for fluid components, with the operator focused on the quantitative and qualitative B-line assessment on the ultrasound screen. As in the case of the previously described ultrasound scans, the assessment and interpretation of the obtained findings may vary depending on the operator’s skill and experience. Therefore, another AI-based technology may be of great support for the assessment of vertical US artifacts. This technology is referred to as Smart B-line (Mindray) and AutoB-line (GE), depending on the manufacturer. A doctor performing a lung scan using this technology positions the ultrasound transducer in exactly the same zones as during conventional imaging. The US machine can assign a diagnostic image to each scanned zone. There are 6, 8, and 12 scanning zones, the number of which is selected by the operator as described Figure 13.

**Figure 14:**
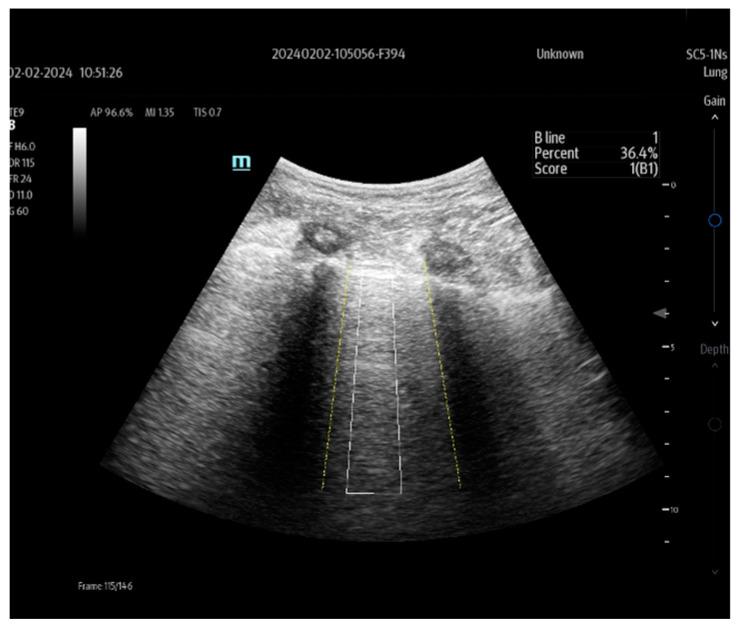
Smart B-line technology (Mindray TEX20; software version 01(01.07.00)).

Automatic identification of the pleural line, the rib acoustic shadow, and the presentation of the scanned zone for the presence of vertical artifacts (B-lines) is the primary support offered by this technology. Once the operator accepts the proposed zone, the B-lines in the selected zone are identified and automatically counted, and the distance between them is measured. In addition to these data, the ultrasound screen also displays information about the B-line area ratio for the entire zone (%), as shown in Figure 14.

**Figure 15:**
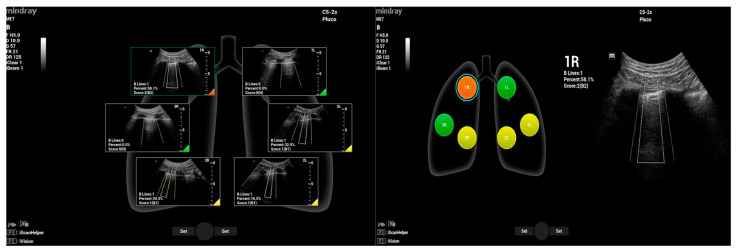
A scoring map and full review—Smart B-line (Mindray TEX20; software version 01(01.07.00)).

Once the scan is complete, the operator can generate a report that will summarize the entire examination along with the scoring of each scanned zone, as shown in Figure 15.

**Figure 16:**
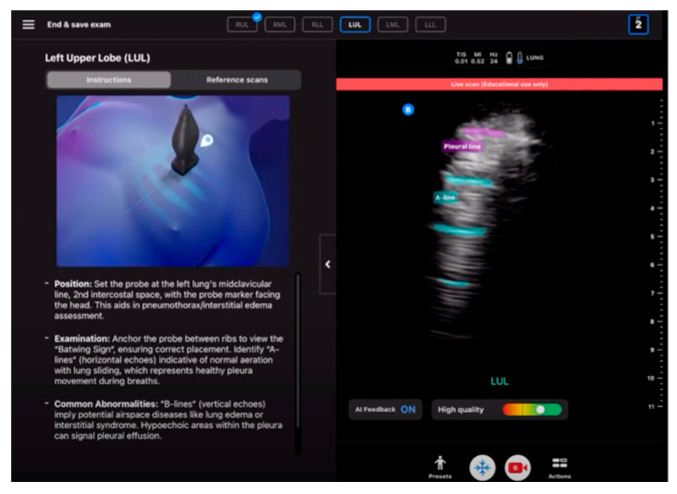
Automatic detection of A-line in lung ultrasound, adapted from (https://www.butterflynetwork.com/iq-plus) with permission publisher 2024 (accessed on 1 January 2024) [29].

More than vertical artifacts can be identified by AI-based technologies in LUS. The technology used in the latest solution from Butterfly Network, Inc. helps identify horizontal artifacts, i.e., the A-lines. As shown in Figure 16, Butterfly ScanLab software employed in the Butterfly iQ+ device identifies horizontal artifacts and the pleura itself during an ultrasound scan, presenting them in appropriate colors.

To summarize, AI-based technologies are used in the ultrasound assessment of the lungs, including patients with COVID-19, and can be very helpful, especially for a less experienced physician, eliminating inter-operator subjectivity of the assessment and providing a large amount of valuable clinical information. Additionally, they are very easy to use [34,35].

**Figure 17:**
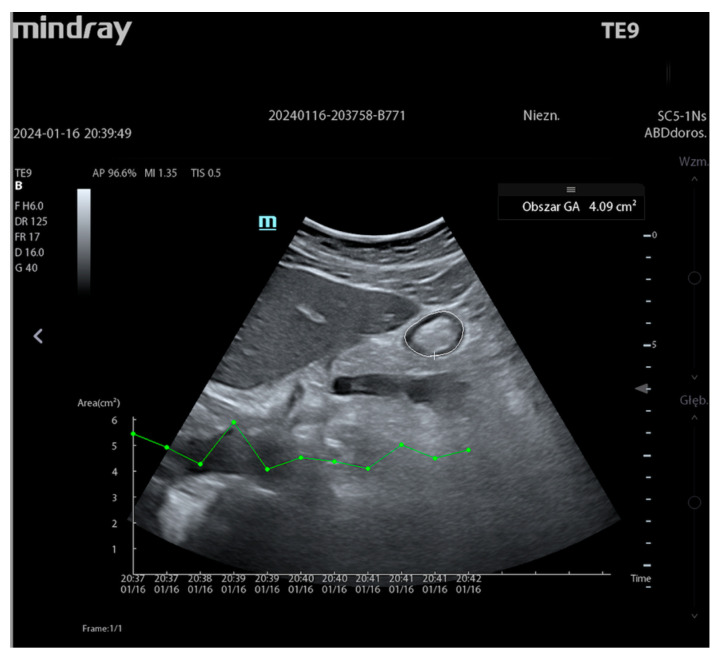
Auto Gastric Antrum (Mindray TEX20; software version 01(01.07.00)).

Auto Gastric Antrum (Mindray), i.e., automatic measurement of gastric contents, is the last technology supported by DL algorithms. In clinical practice, this test is part of the gastric POCUS concept, which is a non-invasive quantitative and qualitative assessment of the gastric contents. Qualitative measurement helps determine whether the stomach is empty or filled with solid or liquid content. Quantitative assessment involves estimating the gastric volume, which is calculated based on the antral cross-sectional area (antral CSA) [36]. This information is very useful for the assessment of the risk of aspiration, which in turn facilitates therapeutic decisions and influences the type of anesthesia or induction techniques. A convex transducer is used, and the patient is placed in two positions—on the back and on the right side. The sagittal view is used as a standard. The operator performs quantitative and qualitative assessment by manually outlining the gastric cardia in the patient placed on the right side. The obtained CSA dimension is directly proportional to the volume, which is ultimately obtained from the data in a mathematical table that takes into account the patient’s age. If the test is repeated several times, it is necessary to archive the data to assess the trend of changes. When Auto Gastric Antrum is used, the operator’s only task is to obtain the correct image accepted by the device. From this moment, the AI-guided ultrasound system will evaluate the image, locate the antrum and automatically outline the CSA, presenting the GA area in cm^2^, as shown in Figure 17.

Since the amount of gastric contents varies over time, it is possible to present the CSA area in the form of a trend line, which is also presented in Figure 17. Therefore, as in each technology described earlier, the automation of diagnostic measurements performed by the AI technology objectifies the obtained results, making them repeatable and avoiding inter-operator variability. In clinical practice, it has become an easy and very useful tool for assessing gastric contents.

**Figure 18:**
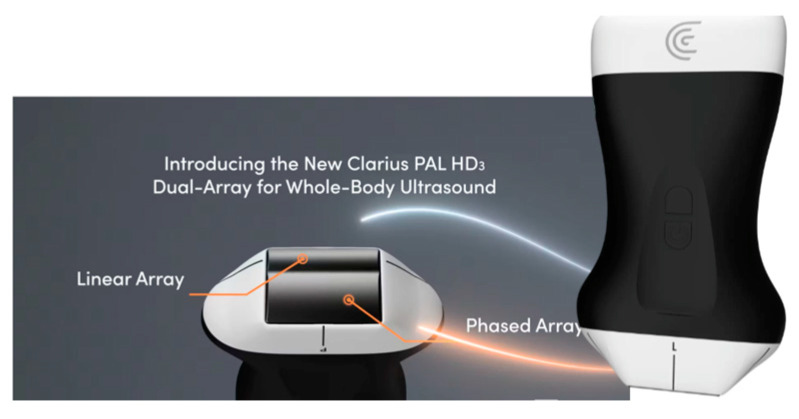
Clarius PAL HD3, adapted from (https://clarius.com/pal-dual-array-ultrasound) with permission publisher 2024 (accessed on 1 January 2024) [37].

Clarius PAL HD3 (Clarius Mobile Health, Vancouver, BC, Canada) is the last intensive-care ultrasonographic solution based on AI, not only in the context of software but also in the context of the optimal use of a single ultrasound transducer to examine the patient’s entire body (Figure 18). This solution allows the use of a single wireless transducer for all ultrasound examinations in an intensive care unit. One transducer, in the shape of a typical phased array transducer, presents an appropriate image as a linear, convex, or phased array transducer, depending on the site where the transducer is applied by the operator. In practice, it works in such a way that if the operator applies the transducer, for example, to the patient’s abdomen, an image typical of a convex transducer will automatically appear on the ultrasound screen, without the need to press any buttons. If the transducer is positioned around the cervical vessels, the image shape will change to the one acquired with a typical linear transducer. If the scan is performed to assess the heart, the presented image will automatically change into one typical of phased array transducers.

If we add to this functionality the fact that the manufacturer also offers the option of voice control for the key functions of the ultrasound system, this solution becomes very practical for the operator.

All the above mentioned AI-based technologies (Table 1) are still at an early stage of implementation, and confirming their usefulness requires extensive research to enable their practical clinical evaluation. Simplified interpretation of images (63%);

- Facilitation of the learning process for ultrasound (59%);
- Streamlined capture of images (47%);
- Enhanced accuracy scanning (42%);
- Quicker diagnosis for accelerated treatment (40%);
- Higher efficiency in workflow (38%).

**Figure 19:**
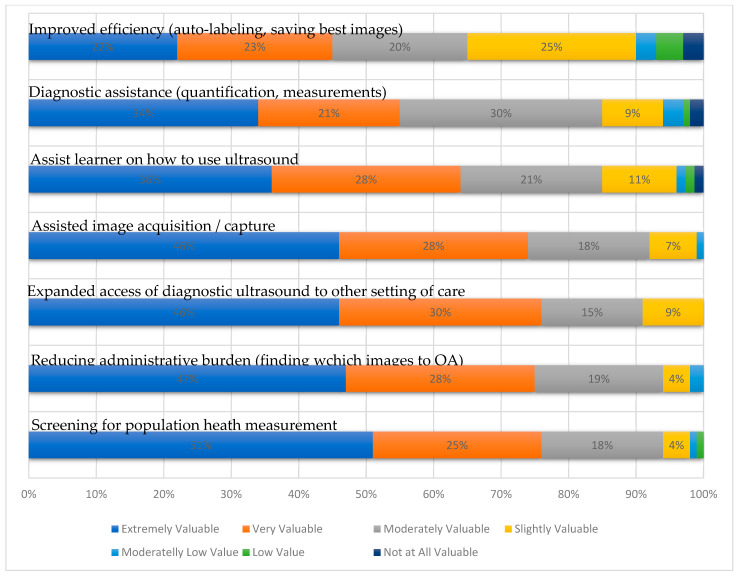
Value added from AI, reproduced from (Exo 2023 Survey Report. Unlocking Point-of-Care Ultrasound) [38].

The benefits of using AI include not only support for beginner ultrasonographers but also for those highly experienced. Survey participants pointed to significant improvements in productivity, with features such as auto-labeling (76%) and daily diagnostic support (75%) of key importance. Most of all, AI reduces the scanning time, which allows a larger number of patients to be examined. Additionally, diagnostic AI support may be used to confirm the initial diagnosis established by experienced clinicians, thus accelerating therapeutic processes in an intensive care unit (Figure 19) [38].

## 3. Conclusions

Introduction of ultrasound to assess a patient’s health status was the first revolution in intensive care. Currently, as shown in this paper, artificial intelligence facilitates ultrasound imaging and measurements crucial for selected areas of intensive care, significantly reducing the workload of medical staff. Introduction of complex systems based on artificial intelligence, which will increase the diagnostic speed and effectiveness, as well as facilitate making complex clinical decisions, will most likely represent another milestone. The complexity of an intensive care unit, the instability and severity of patients’ condition, and the need to combine the medical knowledge of specialists in various medical fields will most likely make it impossible to create independent autonomous systems for assessing patient health status, but the emerging solutions will significantly accelerate the diagnostic and therapeutic path, improving the treatment outcomes of intensive care patients.

## Figures and Tables

**Table 1:** Summary of selected technological and artificial intelligence in ultrasonography in the field of ICU.

AI Technology	Manufacturer	Short Description	Range of Ultrasound Exam
Lvivo	Philips	automatic measurement of stroke volume (SV) and cardiac output (CO)	Ultrasonographic assessment of cardiac function
AutoVTI	GE		
US2.AI	EchoNous		
SmartVTI	Mindray		
SmartEchoVue	Mindray	assessment of views	Whole body
Us2.ai and AI TRO	EchoNous		
Butterfly ScanLab	Butterfly Network		
LVivo Seamless	Philips		
AutoEF	Mindray	calculation of ejection fraction (EF)	Ultrasonographic assessment of cardiac function
RealTimeEF	GE		
US2.AI	EchoNous		
LvivoEF	Philips		
SmartIVC	Mindray	automatic measurement of the inferior vena cava (IVC)	Internal Vena Cava examination
AutoIVC	GE		
Smart B-line	Mindray	automatic identification of the artifacts	Lung examination
Butterfly ScanLab	Butterfly Network		
Auto B-line	GE		
Auto Gastric Antrum	Mindray	automatic measurement of gastric contents	Gastric examination
Clarius PAL HD3	Clarius Mobile Health	1 probe for each examination	Whole body

## Data Availability

Not applicable.
